# Data on physico-chemical characteristics and elemental composition of gray forest soils (Greyzemic Phaeozems) in natural-technogenic landscapes of Moscow brown coal basin

**DOI:** 10.1016/j.dib.2021.106817

**Published:** 2021-01-30

**Authors:** Alexander S. Kostin, Pavel P. Krechetov, Olga V. Chernitsova, Elena V. Terskaya

**Affiliations:** Faculty of Geography, Lomonosov Moscow State University, GSP-1, Leninskie Gory, 119991 Moscow, Russia

**Keywords:** Spoil heaps, Acid mine drainage, Mine subsidence, Soils with technogenic transformation, Physico-chemical soil properties, Elemental composition

## Abstract

Waste rocks material and acid mine drainage (AMD) in sulfur coal mining areas of Moscow brown coal basin lead to significant transformation of landscape components (soils, surface, and groundwaters). Most of the abandoned sulfide-bearing spoil heaps have not been reclaimed and toxic products of their weathering cause the risk of long-term soil contamination. In this article, we report original data on some physico-chemical properties and elemental composition of liquid and solid soil phases, waste dumps and AMD from twо abandoned spoil heaps of the Moscow basin and adjacent territories (the Tula region, Central Russia). Soil samples were collected from each genetic horizon of soil depth profile at sites affected by waste dumps and mine subsidence, as well as at natural sites. Waste material was sampled from the different parts of the spoil heaps. Sampling of AMD was performed in technogenic reservoirs near waste dumps. In displaced liquid phases (by ethanol) from soils and waste dump material, natural superficial waters and AMD pH-value, electrical conductivity (EC), the content and composition of readily soluble salts (by high-performance liquid chromatography (HPLC)), as well as titratable acidity (H^+^and Al^3+^) and, water-soluble Fe (using UV/Vis spectrophotometry) were measured. In bulk soil samples organic carbon (C_org_), exchangeable cations (Cа^2+^, Mg^2+^, H^+^, Al^3+^ in KCl-extracts) and hydrolytic acidity (in CH_3_COONa-extracts) were determined. The obtained data can be used to understand the behavior of сhemical elements in soil profiles polluted by coal mining; the negative impact of mine wastes on soil salinity; when identifying pollution levels of potentially hazardous elements in soils affected by coal mining and for complex remediation of spoil heaps in Moscow brown coal basin.

## Specifications Table

**Table:** 

Subject area	Earth Science, Environmental Chemistry
Specific subject area	Earth Science, Environmental Chemistry, Soil Chemistry, Landscape Geochemistry
Type of data	Raw data, tables and figures
How data were acquired	Potentiometric analysis, conductimetric analysis, high performance liquid chromatography (HPLC), UV/Vis spectrophotometry, X-ray fluorescence (XRF) spectrometry, particle-size analysis. Models: Expert 001 ionometer (Econics Expert, Russia); SevenEasy S30 conductometer (Mettler Toledo, Switzerland); Styer liquid chromatograph (Aquilon, Russia); Odyssey DR 2000 UV/Vis spectrophotometer (Hach, USA); MAKS-GV XRF-fluorescence spectrometer (Spektron, Russia); Olympus Innov-X Delta Professional XRF-fluorescence portable analyzer (Delta-X, USA); Analysette 22 MicroTec plus laser particle sizer (Fritsch, Germany). Data on physico-chemical properties of soils, soil solutions, waste dump material, natural superficial waters, and acid mine drainage (AMD) were received by means of standard techniques.
Data format	Raw
Parameters for data collection	Samples (*n* = 100) of soils, waste dump material, natural superficial waters, and AMD were collected at twо abandoned spoil heaps of Moscow brown coal basin: Smirnovskaya-6 (the key site «Кireevsk») and at Skuratovskaya-6 (the key site «Tula»), and at adjacent territories. Both spoil heaps were sampled for waste dump material. Soil samples were taken from genetic horizons in 8 soil profiles (4 locations at each key site) of natural soils and soils with technogenic transformation. Natural surface waters were sampled at 4 locations (two for each key site). The sampling of AMD was performed from technogenic reservoirs at 3 points (2 locations at the key site «Kireevsk» and 1 location at the key site «Tula», respectively).
Description of data collection	The sampling of soils, waste dump material, natural superficial waters, and AMD was performed at Smirnovskaya-6 (the key site «Кireevsk) and at Skuratovskaya-6 (the key site «Tula») spoil heaps where mining had been stopped in 1986 and 1955, respectively. Soils were sampled in soil pits from each genetic horizon up to the depth of 110–150 cm. The sampled soils were Greyzemic Phaeozems with the technogenic transformation of the soil profile (overlapped by technogenic deposits, under the release of AMD from the spoil heaps or in mine subsidence), as well as natural (reference) Greyzemic Phaeozems. Waste material was sampled at the foothill of the Smirnovskaya-6 and the slope of the Skuratovskaya-6 spoil heaps. Samples of surface waters were taken from the ponds and rivers at a distance of 200–1000 m from the spoils. The sampling of AMD was performed from technogenic reservoirs at the release points near waste dumps. The samples of soils and waste rocks were sieved, air-dried, and were ground to a particle size of 1 mm. The water samples were filtered through 0.45 µm PVDF (MillesHV, Millipore) filters. Samples for the particle-size analysis were pre-treated with 4% Na_4_P_2_O_7_. Soil solutions (liquid phases) were displaced by ethanol (Ishcherekov-Komarova method) [4].
Data source location	Data source location The sampling sites were located in the southern part of Moscow brown coal basin (the Tula Region, Russia). Natural landscapes are watersheds and gentle slopes with deciduous forest, mixed-grass meadows, and fallow lands of the northern part of the Central Russian upland. Prevailing soils are Greyzemic Phaeozems Albic (IUSS Working Group WRB, 2015). Soils affected by abandoned coal mining have signs of the technogenic transformation. GPS coordinates of the sampling locations were as follows: 1. The key site «Kireevsk» KRS-1 53°56′47″ N 37°46′26″ E KRS-2 53°56′47″ N 37°46′18″ E KRS-3 53°56′40″ N 37°46′40″ E KRS-4 53°56′34″ N 37°46′24″ E KRD 53°56′32″ N 37°46′24″ E KRF-1 53°56′2″ N 37°46′28″ E KRF-2 53°56′20″ N 37°46′29″ E KRW-1 53°57′01″ N 37°47′27″ E KRW-2 53°56′57″ N 37°47′28″ E 2. The key site «Tula» TLS-1 54°06′24″ N 37°38′34″ E TLS-2 54°06′27″ N 37°38′30″ N TLS-3 54°06′25.2″ N 37°38′28″ E TLS-4 54°06′28″ N 37°38′29″ E TLD 54°06′25″ N 37°38′26″ E TLF 54°06′29″ N 37°38′29″ E TLW-1 54°06′29″ N 37°38′17″ E TLW-2 54°06′30″ N 37°38′09″ E
Data accessibility	All the data are in this article

## Value of the Data

• First open access complex database on physico-chemical properties of solid (exchangeable cations) and liquid (soil solutions) phases, levels of macro- and microelements and particle size of the transformed soils, waste dumps and acid mine drainage in sulfur coal mining areas of the Moscow brown coal basin in Central European Russia. Reported complex data are the key to understanding the geochemical processes occurring in soils polluted by coal mining.
• The data can be used by researchers to understand migration and accumulation of сhemical elements in soil profiles affected by coal mining as well as to forecast the negative impact of mine wastes on soil salinity status.
• The data obtained might be helpful in the identification of pollution levels and in the geochemical assessment of technogenic anomalies of potentially hazardous elements in the soils affected by coal mining.
• The data may be useful for policy makers to develop programs for complex remediation of spoil heaps and forest-steppe landscapes in the Moscow brown coal basin.

## 1 Data Description

**Fig. 1:**
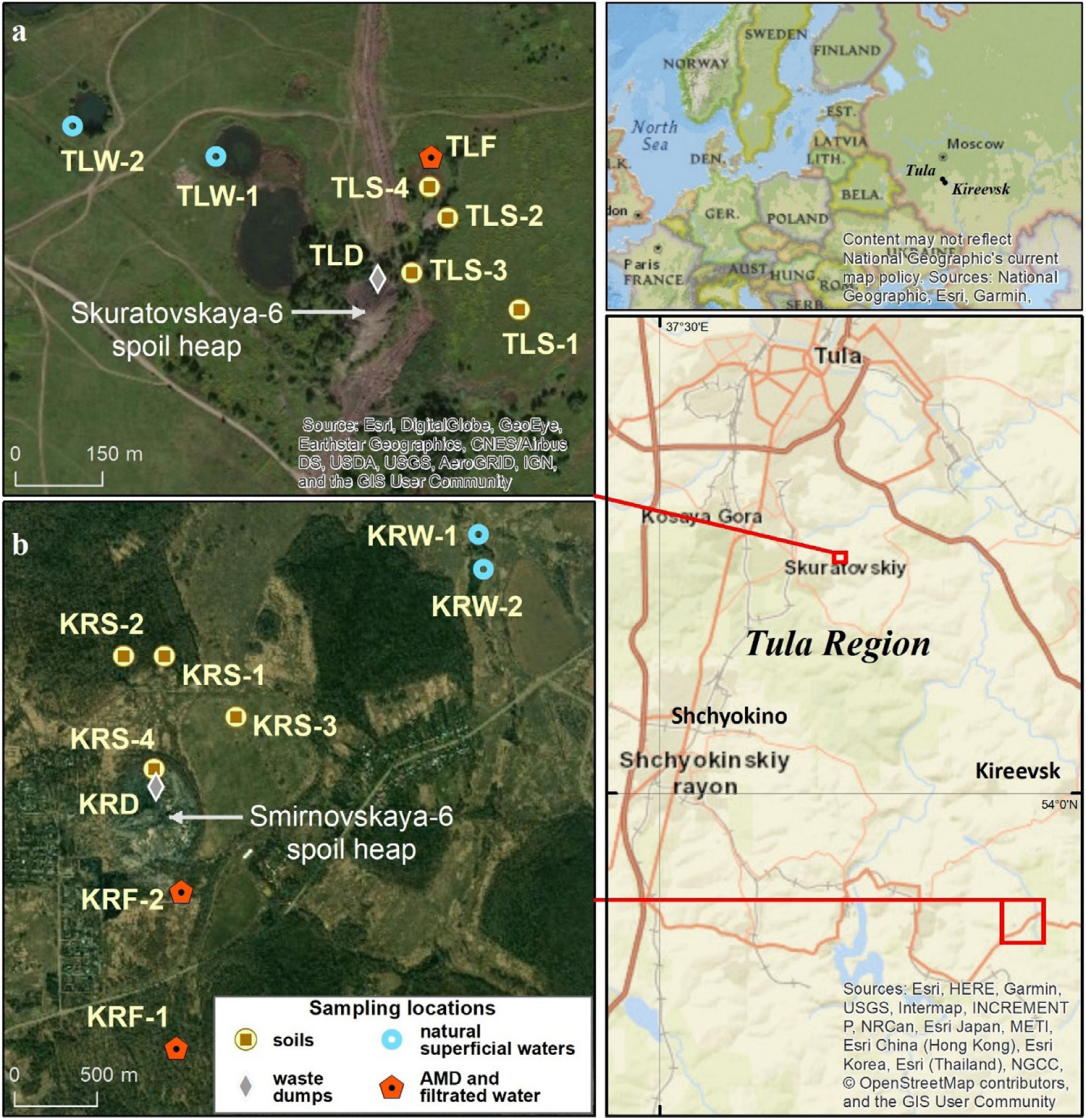
Sampling sites; sampling locations at the key site «Tula» (а) and the key site «Kireevsk» (b). Sample type designations: S-soil, d-waste dumps, W-natural superficial waters, F-acid filtrated waters and AMD. Authors have the right to reuse and reproduce the maps shown in the figures.

**Fig. 2:**
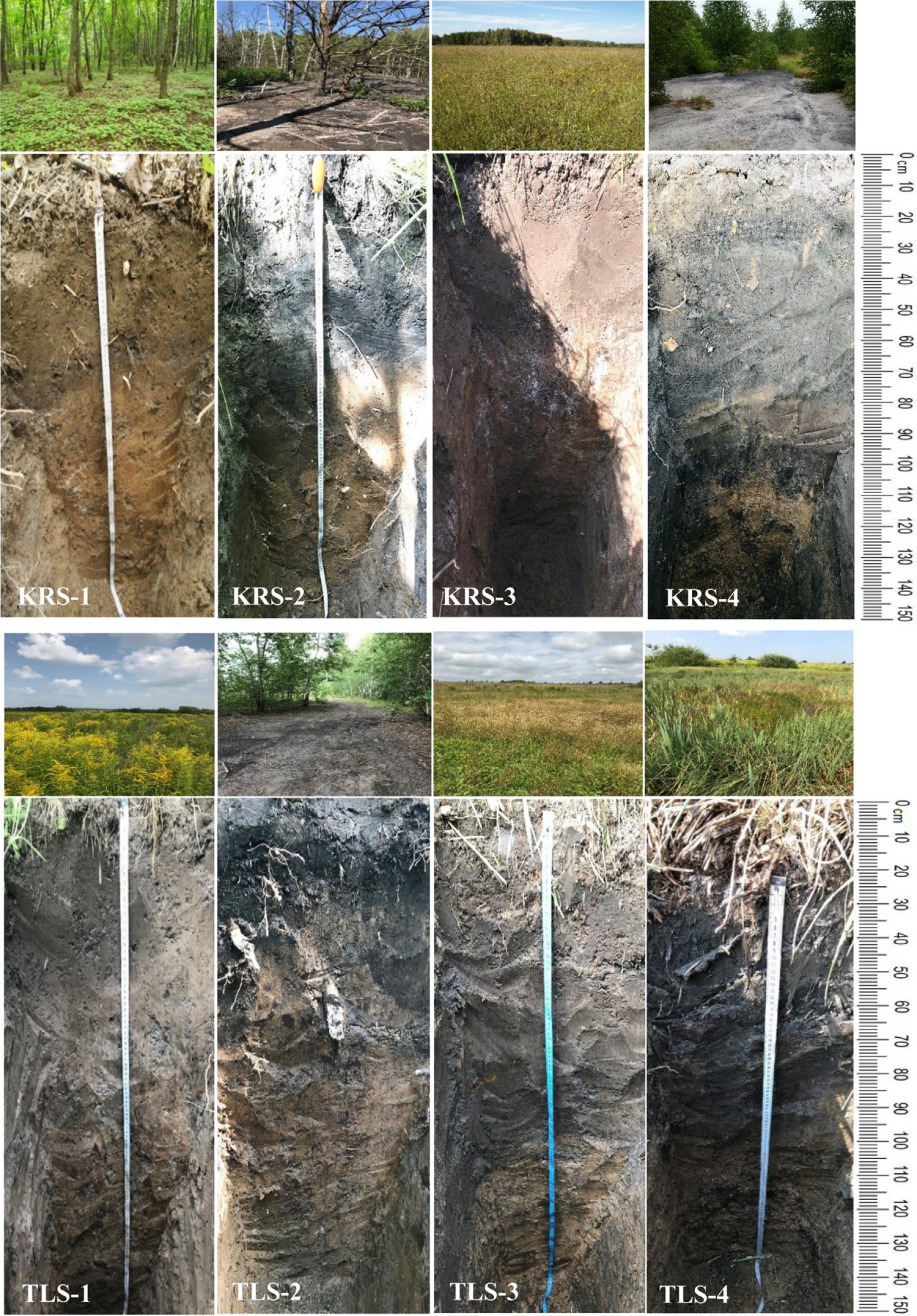
Photos of soil pits. Pit locations are characterized in Table 1. Abbreviated names of places of sampling are the same as given in Fig. 1.

Fig. 1 shows sampling locations of soils, waste dumps, natural superficial waters, and AMD at two key sites. The number of each sampling point is supplemented by a capital letter «S», «D», «F» or «W» («S» for soil, «D» for waste dumps, «F» for AMD (filtrated waters) and «W» for natural superficial waters). Photos of the soil profiles are presented in Fig. 2. Description of the sampling points location and morphological properties of natural soils, and soils with technogenic transformations are given in Table 1. Table 2 contains data on the selected chemical properties (pH value, electrical conductivity (EC), content and composition of readily soluble salts, titratable acidity, water-soluble Fe^2+^ and Fe^3+^) of natural superficial waters and AMD released from the spoil heaps. Data on chemical properties (pH value, readily soluble salts, titratable acidity, water-soluble Fe^2+^ and Fe^3+^) of displaced liquid phases from soils and waste dump material are given in Table 3. Content of organic carbon (C_org_), pH value of KCl-extracts, concentrations of exchangeable cations and hydrolytic acidity of natural soils, and soils with technogenic transformations are shown in Table 4. Data on distribution of five grain-size soil fractions (1000–250, 250–50, 50–10, 10–1 and <1 µm) are presented in Table 5. Concentrations of macroelements (Fe, Si, Al, Ca, Mg, Ti, S, P, K) and microelements (Mn, V, Cr, Ni, Zn, Pb, Sr) in reference and transformed soils of the key site «Kireevsk» are shown in Table 6.

**Table 1:** Description of the locations and morphological properties of natural reference soils, soils with the technogenic transformation, and waste dumps.

Sampling point	Terrain and distance (d) from the waste dump	Soil (IUSS Working Group WRB, 2015)	Horizon	Depth, cm	Description	Land use and vegetation
KRS-1, reference	Gently sloping surface (1–2°), d∼450 m.	Greyzemic Phaeozems Albic	AY(ра)	0–17	gray in color with a brownish shade. friable sandy loam with a lumpy and nutty structure. silica powdering on soil peds.	Forested area. Deciduous forest (*Tilia cordata, Quercus robur, Acer platanoides, Fraxinus excelsior*)
			AEL[hh]	17–22	Whitish-gray with a brown patches in color. Weakly-сompacted sandy loam with a platy and nutty weak structure.	
			BEL[hh]	22–29	Brownish-gray in color with a dark brown tongues. Middle compacted medium textured loam with a nutty structure. Silans and skeletans are sparsely distributed on soil peds. Rusty-brown nodules and spots of Fe and Mn on faces of structural units.	
			BT_1_	29–78	Light-brown in color with humus tongues on cracks. Highly-compacted clay loam with a nutty and prismatic structure. Fine silica bleached powdering on soil peds.	
			BT_2_	78–110	Yellowish-brown in color with ocherous shade. Highly-compacted clay loam with a prismatic and nutty structure. Humus films and clay films on faces of soil peds.	
KRS-2	Dump tailing, plain surface, d∼25 m.	Greyzemic Phaeozems Albic (Carbonic, Gypsic, Novic, Thionic)	W+ТD	0–7	Weakly-developed humus horizon mixed with technogenic deposit.	Industrial site. Low-density projective cover (less than 10%). Vegetation is represented by herbaceous species with undergrowth of *Acer platanoides* and *Betula pendula*.
			ТD*	7–32	Technogenic deposit. Whitish-gray in color with black carbonaceous interlayers. Weakly-compacted loamy sand. Structureless. Numerous inclusions of coal fragments, pyrite concretions.	
			[AY]	32–52	Uneven, dark-gray in color. Highly-compacted sandy clay loam with a prismatic and nutty structure. Yellow patches of pyrite on soil peds and carbonic admixtures in soil mass.	
			[BEL]	34–84	Yellowish-brown in color. Highly-compacted sandy clay loam with a nutty structure. Light-brown clay cutans and dark-gray carbonaceous patches on soil structural units.	
			[BT]	84–110	Dark-brown in color with yellowish shade. Highly-compacted sandy clay loam with a coarsely nutty structure. Soil structural units are covered by bleached silica powder. Carbonaceous particles in cracks and root holes occur.	
KRS-3, reference	Plain surface, d∼450 m.	Greyzemic Phaeozems Albic (Anthric)	P	0–29	Light-gray with a brown shade in color. Weakly-compacted sandy clay loam with an aggregate lumpy and blocky structure. Silica powdering on soil peds оссurs.	Fallow land. Motley-grass meadow (projective cover degree is 80–90%)
			AEL	29–44	Whitish-gray in color with a brown shade. Highly-compacted due to the presence of a plow pan, medium clay loam with a platy weak structure. Soil structural units are covered by bleached skeletans and silans.	
			BEL[hh]	44–64	Light-brown in color with a gray shade. Medium compacted clay loam with a nutty structure. Brown clay cutans and dark-gray films of humus on soil peds. Soil structural units are covered by bleached silica powder.	
			BT_1_	64–97	Brown in color. Highly-compacted loamy clay with a prismatic and nutty structure. Rusty-brown Fe-Mn nodules and dark-brown clay cutans on structural units occur. Soil peds are covered by bleached skeletans and silans.	
			BT_2_	97–132	Yellowish-brown in color. Medium compacted loamy clay with a prismatic and nutty structure. Less pronounced clay cutans on faces of structural units. Ohreous Fe-Mn соncretions and nodules and dark-brown clay cutans on structural units.	
KRS-4	Dump tailing, gently sloping surface (1–2°), d∼350 m.	Greyzemic Phaeozems Albic (Carbonic, Gypsic, Novic, Thionic)	W+TD	0–4	Weakly-developed humus horizon mixed with technogenic deposit	Industrial site. Low-density projective cover (less than 10%). Vegetation is represented by herbaceous species with undergrowth of *Betula pendula*.
			ТD	4–36	Technogenic deposit. Uneven, whitish-gray in color with black carbonaceous interlayers. Weakly-compacted loamy sand. Structureless. Numerous inclusions of coal fragments, weathered rocks, grains of pyrite and single dots of gypsum.	
			[AY+BEL]	36–77	Uneven, pale brown in color with yellow sandy patches. Highly-compacted sandy loam with an aggregate nutty structure. Black carbonaceous films and weak bleached silica powder on faces of soil peds.	
			[BT_1_]	77–89	Uneven, yellowish-brown in color. Highly-compacted medium loam with a weak nutty structure. Rusty-ochreous spots, bluish-whitish patches and carbonaceous particles in soil pore space.	
			[BT_2_]	89–110	Uneven, pale yellowish-brown in color. Highly-compacted loamy clay with a weak prismatic structure. Ochreous and bluish spots of ferrous and ferric Fe in soil pore space.	
TLS-1, reference	Plain surface, d∼230 m.	Greyzemic Phaeozems Albic	AYpa	0–23	Light-gray in color. Friable medium silt loam with weak lumpy structure. Silica powdering on soil peds.	Fallow land. Motley-grass meadow (projective cover is 70–80%)
			AEL	23–32	Uneven, light-gray in color with brownish and whitish patches.Weakly-compacted sandy loam due to the presence of a plow pan with platy and lumpy structure. Intensive silica powdering on soil peds.	
			BEL	32–52	Brown in color with gray patches. Weakly-compacted medium textured loam with platy and lumpy structure. Nest-like inclusions of silica powder in soil mass.	
			BT_1_	52–85	Вrown in color. Highly-compacted loamy clay with a prismatic and nutty structure. Rare Fe-Mn nodules and dark-brown clay cutans on structural units. Soil peds are covered by bleached skeletans and silans.	
			BT_2_	85–116	Yellowish-brown in color. Highly-compacted loamy clay with a weak prismatic structure. Сlay cutans on faces of structural units.	
TLS-2	Dump tailing, plain surface, d∼75 m.	Greyzemic Phaeozems Albic (Carbonic, Gypsic, Novic, Thionic)	W+TD	0–7	Weakly-developed humus horizon mixed with technogenic deposit.	Industrial site. Low-density projective cover (less than 10%). Vegetation is represented by herbaceous species with undergrowth of and *Betula pendula* and *Populus tremula*)
			TD+AY	7–31	Technogenic deposit mixed with humus horizon of soils. Uneven, whitish-gray in color with a brown shade. Weakly-сompacted loamy sand. Structureless. Numerous inclusions of coal fragments, pyrite concretions and single gypsum crystals.	
			[BEL]	31–64	Uneven, pale brown in color with whitish and gray patches. Compacted sandy loam with weak platy and nutty structure. Soil structural units are covered by bleached skeletans and carbonaceous films.	
			[BT_1_]	64–90	Uneven, yellowish-brown in color. Highly-compacted medium loam with a weak nutty structure. Soil peds are covered by bleached skeletans and carbonaceous films.	
			[BT_2_]	90–122	Uneven, pale yellowish-brown in color. Highly-compacted loamy clay with a weak prismatic and blocky structure. Rusty-brown Fe-Mn nodules on structural units.	
TLS-3	Mine subsidence (without excessive moisture), gently sloping surface (1–2°), *d* = 90 m	Greyzemic Phaeozems Albic (Gleyic, Technic)	AUpa	0–22	Dark-gray in color. Friable sandy loam with crumb-like and lumpy structure. Soil peds are covered by rare bleached skeletans.	Fallow land. Projective cover is 80–90% (hydrophilious herbaceous species).
			AUe	22–47	Dark-gray in color with whitish shade.Weakly-compacted sandy loam with crumb-like and lumpy structure. Silica powdering on soil peds.	
			BELg	47–68	Brownish-gray in color with bluish shade. Middle compacted medium textured loam with a lumpy and nutty structure. Weak silica powdering on soil structural units. Brown clay cutans and dark-gray films of humus on cracks and root holes.	
			BTg_1_	68–98	Yellowish-brown in color with bluish shade. Highly-compacted loamy clay with a nutty structure. Weak silica powdering on soil peds. Rusty-brown Fe-Mn nodules, brownish-bluish clay and dark-gray humus films on structural units.	
			BTg_2_	98–132	Yellowish-brown in color with bluish and steel-like shade. Highly-compacted loamy clay with a coarsely nutty and prismatic structure. Signs of gleying (ohreous and bluish patches) in soil mass. Rusty-brown Fe-Mn nodules on structural units.	
TLS-4	Mine subsidence сonnected with dump tailing (waterlogged area), gently sloping surface (1–2°), *d* = 320 m	Greyzemic Phaeozems Albic (Gleyic, Carbonic, Gypsic, Thionic, Technic)	AUh	0–8	Organogenic horizon. Brownish-dark-gray in color with a steel-like shade. Weakly-compacted sandy loam with а lumpy structure. Soil mass consist of partially decomposed humus and muck material.	Industrial site. Projective cover is 80–90% (hydrophilious herbaceous species).
			AUe,g	8–42	Brownish-gray in color with a bluish shade. Weakly-compacted sandy loam with а lumpy and platy structure. Rare silica powdering on soil peds.	
			BELg	42–56	Grayish-brown in color with a bluish shade. Сompacted medium-textured loam with а nutty structure. Rusty-brown and black Fe-Mn nodules in soil mass.	
			BTG_1_	56–73	Uneven, bluish-brown in color with dark-gray and yellow patches. Highly-compacted medium-textured loam with а prismatic and blocky structure. Signs of gleying (ohreous and bluish patches) in soil mass. Rusty-brown Fe-Mn nodules on structural units.	
			BTG_2_	73–117	Uneven, bluish-brown in color with ochreous and yellow patches. Highly-compacted medium-textured loam with а blocky structure. Signs of gleying (ohreous and bluish patches) in soil mass. Rusty-brown Fe-Mn nodules on structural units.	
KRD	Foothill of the spoil heap	0–100			Pirogenically-transformed waste-rock material comprising of different grain size particles with fragments of coal and concretions of pyrite.	Waste dump. Single undergrowth of *Betula pendula*.
TLD	Middle part of slope of the spoil heap	0–100			Waste-rock material comprising of different grain size particles with fragments of coal and concretions of pyrite.	Waste dump. No vegetation.

TD* - technogenic deposit.

**Table 2:** Chemical properties (pH-value, EC, the composition of readily soluble mineral salts, titratable acidity, water-soluble Fe) of natural superficial waters, and AMD.

				Concentration of readily soluble ions, mmol_c_/dm^−3^							Titratable acidity, mmol_c_/dm^−3^		Fe, mmol_c_/dm^−3^	

Sampling point	рН	EC, μS cm^−1^	Total mineralization, mg L^−1^	HCO_3_^−^	Cl^−^	SO_4_^2−^	Ca^2+^	Mg^2+^	K^+^	Na^+^	H^+^	Al^3+^	Fe^2+^	Fe^3+^
KRF-1	3.6	2360	1968.34	<0.01	0.15	3.14	5.21	1.29	0.03	0.32	0.75	7.80	<0.01	<0.01
KRF-2	4.3	1624	1249.96	<0.01	0.06	16.03	18.86	0.93	0.09	0.08	0.37	3.07	<0.01	0.68
KRW-1	8.0	667	286.56	1.13	0.27	1.97	4.48	0.60	0.03	0.26	0.49	0.32	<0.01	<0.01
KRW-2	8.1	300	164.76	1.19	0.08	0.09	2.91	0.35	0.08	0.18	0.11	0.01	<0.01	<0.01

TLF	5.3	998	702.17	0.07	0.18	9.29	10.69	1.21	0.13	0.49	0.20	0.01	<0.01	<0.01
TLW-1	7.3	518	314.22	0.98	0.28	2.77	3.15	1.03	0.24	1.13	0.13	<0.01	<0.01	<0.01
TLW-2	7.1	517	306.63	0.77	0.31	3.05	2.76	1.02	0.25	1.06	0.15	<0.01	<0.01	<0.01

**Table 3:** Chemical properties (pH-value, the composition of readily soluble mineral salts, titratable acidity, water-soluble Fe) of displaced liquid phases from soils and waste dumps.

						Composition of readily soluble salts, mmol_c_/dm^−3^							Titratable acidity, mmol_c_/dm^−3^		Fe, mmol_c_/dm^−3^	

Sampling point	Horizon	Depth, cm	рН	EC, μS cm^−1^	Total mineralization, mg L^−1^	HCO_3_^−^	Cl^−^	SO_4_^2−^	Ca^2+^	Mg^2+^	K^+^	Na^+^	H^+^	Al^3+^	Fe^2+^	Fe^3+^
KRD	–	0–100	2.6	5940	3543.29	<0.01	0.41	52.08	15.71	0.75	0.06	0.79	42.20	19.72	2.72	<0.01
KRS-1	AY(ра)	0–17	6.4	291	234.20	1.12	0.18	0.19	2.38	0.34	0.09	0.16	0.31	<0.01	<0.01	<0.01
	AEL[hh]	17–22	6.9	101	110.41	0.96	0.14	0.19	0.94	0.15	<0.01	0.23	0.14	<0.01	<0.01	<0.01
	BEL[hh]	22–29	6.6	78	104.04	1.06	0.11	0.14	0.92	0.13	<0.01	0.15	0.27	<0.01	<0.01	<0.01
	BT_1_	29–78	5.3	69	78.56	0.78	0.13	0.08	0.67	0.10	<0.01	0.12	0.42	0.03	<0.01	<0.01
	BT_2_	78–110	6.3	43	72.18	0.80	0.09	0.06	0.57	0.08	<0.01	0.08	0.26	<0.01	<0.01	<0.01

KRS-2	W+TD*	0–7	3.5	1582	1749.74	<0.01	0.02	24.31	13.00	0.11	0.16	0.22	0.96	11.11	0.05	0.01
	ТD	7–32	3.7	993	987.71	0.04	0.12	13.65	7.00	0.43	0.06	0.48	0.54	5,96	0.08	<0.01
	[AY]	32–52	4.1	1539	1552.64	0.16	0.09	21.92	20.60	1.57	0.12	0.16	0.50	1.54	<0.01	0.01
	[BEL]	34–84	3.9	1277	1223.00	0.20	0.11	17.21	13.10	0.82	<0.01	0.29	0.65	3.60	<0.01	0.02
	[BT]	84–110	4.1	1062	1130.04	0.50	0.22	15.75	15.00	1.98	<0.01	0.28	0.45	<0.01	<0.01	<0.01

KRS-3	P	0–29	6.8	407	278.16	2.32	0.19	0.30	4.44	0.39	0.01	0.18	0.23	0.01	<0.01	<0.01
	AEL	29–44	5.7	348	221.39	1.45	0.21	0.21	4.08	0.59	0.01	0.25	1.15	0.01	0.03	<0.01
	BEL[hh]	44–64	6.0	51	48.88	0.34	0.10	0.12	0.53	0.09	<0.01	0.14	0.22	0.01	0.02	<0.01
	BT_1_	64–97	5.4	83	58.69	0.24	0.11	0.26	0.71	0.12	0.01	0.17	0.41	0.08	<0.01	<0.01
	BT_2_	97–132	5.4	56	54.80	0.23	0.14	0.20	0.74	0.11	0.02	0.19	0.51	0.01	<0.01	<0.01

KRS-4	W+TD	0–7	4.0	220	158.71	0.04	0.26	1.23	1.20	0.13	0.29	0.10	9.42	0.24	<0.01	0.01
	TD+AY	7–31	3.7	432	506.03	<0.01	0.45	4.56	8.24	0.29	0.13	0.24	8.92	1.98	<0.01	0.04
	[BEL]	31–64	3.3	2420	2564.67	<0.01	0.78	34.90	16.50	1.14	0.14	0.61	2.80	18.20	<0.01	0.12
	[BT_1_]	64–90	3.7	1033	1969.55	<0.01	0.37	26.58	12.15	0.78	0.05	0.15	2.00	15.20	<0.01	<0.01
	[BT_2_]	90–122	3.9	1507	1567.25	<0.01	0.17	21.33	9.31	0.65	0.13	0.23	0.80	12.10	<0.01	0.02
TLD	–	0–100	2.3	21,400	21,408.2	<0.01	<0.01	250.0	360.0	9.75	0.04	10.83	591.8	34.20	2.39	3.07

TLS-1	AYpa	0–23	7.3	196	116.40	0.83	0.09	0.23	1.98	0.20	0.14	0.01	0.17	0.08	0.01	0.03
	AEL	23–32	5.5	86	91.57	0.41	0.16	0.53	1.14	0.19	0.13	0.02	1.09	0.13	0.01	0.01
	BEL	32–52	5.8	40	43.78	0.18	0.13	0.25	0.53	0.10	0.07	0.01	0.28	<0.01	<0.01	0.01
	BT_1_	52–85	6.0	62	51.31	0.25	0.13	0.22	0.68	0.12	0.15	0.01	0.21	<0.01	<0.01	<0.01
	BT_2_	85–116	5.2	56	47.70	0.13	0.27	0.28	0.49	0.10	0.12	0.01	0.38	<0.01	<0.01	<0.01
TLS-2	W+TD	0–7	3.9	2360	1967.39	<0.01	2.79	23.25	29.26	2.27	0.41	1.43	1.51	3.22	0.03	0.02
	TD+AY	7–31	4.1	2810	2338.81	0.02	2.26	27.92	33.15	2.41	0.47	1.20	0.89	6.59	<0.01	<0.01
	[BEL]	31–64	4.2	1508	1605.47	0.02	3.16	17.58	17.68	2.01	0.10	0.90	0.62	8.86	<0.01	<0.01
	[BT_1_]	64–90	4.3	1609	1620.84	0.02	3.20	18.08	17.86	2.50	<0.01	0.81	0.38	8.50	<0.01	<0.01
	[BT_2_]	90–122	4.3	1448	1560.64	0.02	2.79	17.73	14.56	2.95	<0.01	0.73	0.84	9.71	<0.01	<0.01

TLS-3	AUpa	0–22	6.7	1470	1082.75	0.38	0.35	13.81	16.76	1.67	0.08	0.99	0.36	<0.01	<0.01	0.01
	AUe	22–47	5.7	1327	949.02	2.21	0.25	9.40	15.00	1.47	0.28	0.67	2.32	<0.01	<0.01	0.08
	BELg	47–68	7.0	714	498.82	0.48	0.27	5.83	7.80	0.84	0.06	0.44	0.16	<0.01	<0.01	<0.01
	BTg_1_	68–98	7.1	87	128.74	0.28	0.23	1.25	1.51	0.21	0.01	0.22	0.18	<0.01	<0.01	<0.01
	BTg_2_	98–132	7.1	87	115.45	0.25	0.18	1.13	1.44	0.22	0.01	0.20	0.16	<0.01	<0.01	<0.01

TLS-4	AUh	0–8	3.9	481	410.99	<0.01	5.36	0.19	5.85	0.27	0.05	0.15	3.58	0.55	0.01	0.02
	AUe,g	8–42	4.0	581	641.63	<0.01	7.93	0.22	6.55	0.88	0.03	0.14	2.95	3.63	0.02	0.03
	BELg	42–56	4.0	2800	2056.70	<0.01	25.44	0.29	27.62	1.91	0.04	0.29	1.18	8.87	0.01	0.03
	BTG_1_	56–73	4.0	2490	2304.12	<0.01	28.54	0.34	30.00	2.09	0.05	0.30	0.98	10.60	<0.01	0.02
	BTG_2_	73–117	4.5	1631	1670.62	0.02	21.40	1.06	17.18	4.59	0.01	0.92	0.42	6.71	<0.01	<0.01

TD*- technogenic deposit.

**Table 4:** C_org_, exchangeable cations, and hydrolytic acidity in soils and waste dumps.

Sampling point	Horizon	Depth, cm	рН_KCl_	C_org_, %	Cа^2+^	Mg^2+^	H^+^	Al^3+^	Fe^2+^	Fe^3+^	
					cmol_c_ kg^−1^				cmol_c_ kg^−1^		Hydrolytic acidity, cmol_c_ kg^−1^
KRD	–	0–100	2.6	25.71	1.65	0.83	1.74	2.74	0.67	0.11	41.76
KRS-1	AY(ра)	0–17	5.7	1.43	9.50	2.88	0.06	<0.01	ND*	ND	2.10
	AEL[hh]	17–22	5.8	0.94	12.75	4.00	0.06	<0.01	ND	ND	2.64
	BEL[hh]	22–29	5.3	0.45	15.13	4.25	0.08	0.01	ND	ND	2.25
	BT_1_	29–78	4.8	0.35	17.00	4.13	0.08	0.18	ND	ND	1.78
	BT_2_	78–110	5.0	0.31	16.75	4.25	0.05	0.14	ND	ND	2.00

KRS-2	W+ТD	0–7	3.4	3.16	1.00	0.75	0.05	2.45	ND	ND	7.59
	ТD*	7–32	3.4	4.18	1.13	0.88	0.20	4.91	ND	ND	13.15
	[AY]	32–52	3.9	1.90	9.13	2.38	0.09	4.23	ND	ND	7.04
	[BEL]	34–84	4.2	0.38	12.30	2.80	0.10	2.10	ND	ND	1.88
	[BT]	84–110	4.8	0.37	18.75	3.38	0.05	0.15	ND	ND	1.83

KRS-3	P	0–29	6.3	2.56	14.30	2.89	0.05	<0.01	<0.01	<0.01	1.13
	AEL	29–44	6.2	1.18	18.29	3.44	0.05	<0.01	<0.01	0.05	1.05
	BEL[hh]	44–64	5.1	0.72	16.09	6.46	0.08	0.13	<0.01	<0.01	2.34
	BT_1_	64–97	4.8	0.58	15.81	6.05	0.06	0.11	<0.01	<0.01	2.56
	BT_2_	97–132	4.8	0.53	16.50	7.70	0.09	0.14	<0.01	0.01	2.51

KRS-4	W+TD	0–7	3.5	3.47	0.63	0.25	0.10	3.65	0.23	0.02	10.09
	TD+AY	7–31	3.6	4.52	0.88	0.63	0.36	8.51	0.21	0.01	14.31
	[BEL]	31–64	3.4	9.55	11.50	2.50	0.90	3.06	0.20	<0.01	20.76
	[BT_1_]	64–90	3.9	1.24	2.13	1.25	4.69	12.28	<0.01	<0.01	10.25
	[BT_2_]	90–122	3.7	0.35	2.88	1.63	4.44	12.93	0.01	0.01	15.91
TLD	–	0–100	2.3	13.21	8.94	19.11	14.61	7,18	0.64	<0.01	38.35

TLS-1	AYpa	0–23	6.2	1.58	14.99	1.79	0.04	<0.01	<0.01	<0.01	2.11
	AEL	23–32	6.0	0.66	13.75	3.30	0.06	<0.01	<0.01	<0.01	1.89
	BEL	32–52	5.5	0.33	15.26	3.03	0.08	0.09	<0.01	<0.01	2.26
	BT_1_	52–85	5.0	0.27	17.46	4.13	0.06	0.39	<0.01	<0.01	2.35
	BT_2_	85–116	5.1	0.25	17.46	5.36	0.08	0.25	<0.01	<0.01	2.15

TLS-2	W+TD	0–7	3.5	9.05	11.00	5.78	1.81	3.18	0.49	0.03	21.85
	TD+AY	7–31	3.9	1.77	7.01	0.82	0.10	5.30	0.26	<0.01	11.01
	[BEL]	31–64	4.0	1.34	0.96	11.28	0.09	5.11	0.09	0.03	10.24
	[BT_1_]	64–90	4.3	0.44	15.68	7.98	0.08	2.64	0.04	<0.01	4.39
	[BT_2_]	90–122	4.9	0.22	11.00	10.31	0.04	0.44	<0.01	<0.01	2.28

TLS-3	AUpa	0–22	5.3	2.81	14.16	4.95	0.14	0.01	<0.01	<0.01	3.88
	AUe	22–47	6.4	1.15	14.58	3.85	0.05	<0.01	<0.01	<0.01	1.31
	BELg	47–68	6.4	0.66	17.74	4.26	0.10	<0.01	<0.01	<0.01	1.19
	BTg_1_	68–98	6.4	0.37	20.90	6.05	0.09	<0.01	<0.01	<0.01	1.08
	BTg_2_	98–132	6.3	0.17	22.28	0.14	0.11	<0.01	<0.01	<0.01	1.29

TLS-4	AUh	0–8	4.1	5.24	7.43	2.20	0.14	5.78	0.04	<0.01	15.26
	AUe,g	8–42	4.0	1.20	4.26	1.38	0.08	6.10	0.04	<0.01	10.99
	BELg	42–56	3.6	0.56	4.95	2.06	0.45	10.69	0.05	0.04	12.26
	BTG_1_	56–73	3.9	0.26	4.95	2.48	1.36	11.06	<0.01	<0.01	12.80
	BTG_2_	73–117	4.2	0.20	8.53	3.16	0.21	10.05	<0.01	0.01	9.95

TD* - technogenic deposit, ND* - not detected

**Table 5:** Distribution of particle fractions in soils.

			Particle size (mm), %					
Sampling point	Horizon	Depth, cm	250–1000	50–250	10–50	5–10	1–5	<1
KRS-1	AY(ра)	0–17	0.0	2.9	51.6	13.9	24.7	6.9
	AEL[hh]	17–22	0.0	1.4	52.2	14.3	24.3	7.8
	BEL[hh]	22–29	0.0	1.4	52.1	14.2	24.1	8.2
	BT_1_	29–78	0.0	0.4	50.0	14.8	25.6	9.2
	BT_2_	78–110	0.0	0.4	50.0	14.8	25.6	9.2

KRS-2	W+TD	0–7	0.0	8.1	46.1	13.1	24.9	7.8
	ТD*	7–32	0.0	2.8	53.2	13.3	23.8	6.9
	[AY]	32–52	0.0	0.0	45.0	16.8	30.2	8.0
	[BEL]	34–84	0.0	0.0	46.7	16.4	27.2	9.7
	[BT]	84–110	0.0	0.0	43.0	16.8	30.2	10.0

KRS-3	P	0–29	0.0	1.9	51.5	13.9	26.1	6.6
	AEL	29–44	0.0	2.3	54.9	13.5	21.9	7.4
	BEL[hh]	44–64	0.0	1.0	51.5	14.2	24.9	8.4
	BT_1_	64–97	0.0	1.1	50.7	14.3	25.0	8.9
	BT_2_	97–132	0.0	0.8	47.9	15.1	27.0	9.2

KRS-4	W+TD	0–7	0.0	1.1	49.3	15.4	26.8	7.4
	TD+AY	7–31	0.0	16.0	42.9	13.3	21.8	6.0
	[BEL]	31–64	0.0	1.7	46.7	16.0	27.5	8.1
	[BT_1_]	64–90	0.0	2.3	54.4	12.6	23.4	7.3
	[BT_2_]	90–122	0.0	0.0	46.1	15.6	29.1	9.2

TLS-1	AYpa	0–23	0.0	1.2	50.6	15.3	26.8	6.1
	AEL	23–32	0.0	2.8	55.3	13.3	22.0	6.6
	BEL	32–52	0.0	2.0	55.2	12.9	22.3	7.6
	BT_1_	52–85	0.0	2.1	54.6	12.8	22.7	7.8
	BT_2_	85–116	0.0	1.4	49.4	14.0	26.2	9.0

TLS-2	W+TD	0–7	5.8	23.4	41.2	8.4	15.4	5.8
	TD+AY	7–31	0.0	2.4	50.8	14.3	25.9	6.6
	[BEL]	31–64	0.0	1.7	53.1	13.0	24.2	8.0
	[BT_1_]	64–90	0.0	2.6	53.1	13.1	23.6	7.6
	[BT_2_]	90–122	0.0	2.6	53.8	12.4	23.2	8.0

TLS-3	AUpa	0–22	0.2	9.2	51.0	13.4	21.8	4.4
	AUe	22–47	0.0	1.5	50.9	14.5	26.3	6.8
	BELg	47–68	0.0	1.9	53.1	13.5	24.1	7.4
	BTg_1_	68–98	0.0	1.2	44.6	14.0	30.0	10.2
	BTg_2_	98–132	0.0	1.2	45.1	13.9	29.7	10.1

TLS-4	AUh	0–8	0.0	2.3	46.9	15.1	26.9	8.8
	AUe,g	8–42	0.0	2.2	53.0	13.4	24.2	7.2
	BELg	42–56	4.8	4.2	52.2	11.3	20.8	6.7
	BTG_1_	56–73	0.0	1.8	53.2	12.5	24.2	8.3
	BTG_2_	73–117	0.0	1.4	51.1	13.2	25.8	8.5

TD* - technogenic deposit.

**Table 6:** Concentrations of the selected macro- and microelements in soils.

			Macroelements, %									Мicroelements, mg kg^−1^						
Sampling point	Horizon	Depth, cm	Fe	Si	Al	Ca	Mg	Ti	S	P	K	Mn	V	Cr	Ni	Zn	Pb	Sr
KRS-1	AY(ра)	0–17	4.87	34.16	6.53	0.62	0.47	0.62	<0.01	0.08	1.93	1307.1	83.52	89.12	55.05	63.28	25.66	80.84
	AEL[hh]	17–22	3.20	36.68	6.74	0.66	0.47	0.59	<0.01	0.05	2.19	708.9	85.95	92.17	62.15	58.46	39.17	128.4
	BEL[hh]	22–29	5.11	32.98	8.52	0.55	0.61	0.67	<0.01	0.05	1.88	1992.1	109.3	111.8	70.75	67.31	57.82	99.88
	BT_1_	29–78	3.82	33.95	8.17	0.70	0.66	0.61	<0.01	0.05	2.13	576.4	98.46	101.4	60.53	68.07	53.89	123.5
	BT_2_	78–110	3.22	34.72	7.05	0.95	0.55	0.56	<0.01	0.06	2.04	709.5	89.14	94.32	58.97	68.72	21.74	138.9

KRS-2	W+ТD	0–7	0.94	39.16	6.08	0.12	0.10	0.53	0.50	0.02	0.63	38.54	76.28	63.54	26.52	49.84	69.00	7819
	ТD*	7–32	0.97	38.86	5.97	0.12	0.10	0.49	0.30	0.03	0.67	48.13	68.02	63.22	31.02	51.16	74.21	78.35
	[AY]	32–52	3.26	35.69	7.53	0.54	0.46	0.60	0.40	0.05	2.01	751.8	93.37	90.09	56.49	75.40	50.82	136.4
	[BEL]	34–84	3.55	34.93	7.87	0.71	0.58	0.62	0.20	0.04	2.07	556.7	98.24	100.9	66.56	61.04	53.86	136.2
	[BT]	84–110	3.83	34.06	8.22	0.72	0.64	0.62	<0.01	0.05	2.10	563.6	99.98	112.2	61.76	66.28	51.14	128.1

KRS-3	P	0–29	1.80	35.56	6.83	0.50	0.57	0.38	<0.01	<0.01	1.55	907.0	77.00	71.00	41.00	56.00	18.30	110.0
	AEL	29–44	2.47	36.68	6.94	0.45	0.58	0.38	<0.01	<0.01	1.65	389.0	76.00	73.00	39.00	53.00	20.00	105.0
	BEL[hh]	44–64	2.87	33.98	8.72	0.41	0.68	0.40	<0.01	<0.01	1.68	382.0	76.00	63.00	38.00	61.00	16.00	96.00
	BT_1_	64–97	2.90	35.85	8.17	0.42	0.66	0.41	<0.01	<0.01	1.64	416.0	77.00	72.00	36.00	55.00	18.00	100.0
	BT_2_	97–132	2.92	35.42	7.05	0.42	0.62	0.40	<0.01	<0.01	1.63	422.0	81.00	73.00	42.00	58.00	18.00	97.00

KRS-4	W+TD	0–7	1.27	36.19	699	0.14	0.11	0.53	0.40	0.04	0.75	48.94	79.70	67.00	29.04	61.83	64.78	75.21
	TD+AY	7–31	0.79	38.92	5.60	0.12	0.06	0.47	0.50	0.03	0.54	39.72	67.81	62.81	22.44	49.35	60.04	78.03
	[BEL]	31–64	0.71	36.42	7.72	0.18	0.08	0.57	0.60	0.04	0.79	54.38	89.82	70.67	54.87	88.95	118.3	142.5
	[BT_1_]	64–90	2.52	38.53	6.64	0.37	0.37	0.64	<0.01	0.05	2.11	622.5	90.14	81.35	46.43	87.70	61.29	146.0
	[BT_2_]	90–122	3.63	34.68	8.46	0.33	0.52	0.63	0.20	0.05	2.03	455.9	101.9	104.9	59.71	95.95	54.71	125.4

TD* - technogenic deposit.

## 2 Experimental Design, Materials and Methods

### 2.1 Dataset area and objects

The dataset area is situated in the northern part of Central Russian upland and belongs to the southern part of Moscow brown coal basin (the Tula Region, Russia). Watersheds and gentle slopes are occupied by deciduous forests with lime, maple and oak and mixed-grass meadows.

Natural soils of the dataset area are Greyzemic Phaeozems Albic [10] (gray forest soils in Russian classification [3]), silty, heavy loamy on mantle loams. Because of the high percentage of ploughed land (up to 70%) arable and post-arable soils on fallow lands are widespread. In karst sinkholes and local depressions, Gleyic Phaeozems prevail.

Due to the technology of underground mining, conical spoil heaps of waste rocks 40–60 m high were formed on the land surface. Spoils of the Moscow brown coal basin comprise of iron sulfide-bearing carbonaceous black greasy clays with kaolinitic clays, brown coal layers, loams, sandy loams, and quartz sands, as well as pyrite crystals with СаСО_3_ (calcite) and FeCO_3_ (siderite) impurities in clays [8].

The weathering of the spoil heaps led to the formation of deluvial-proluvial tailings of sandy-clay gangue with a high content of sulfides (mainly pyrite and marcasite), as well as organic carbon of coal origin. Technogenic deposits that overlap soils could be up to several dozen centimeters in thickness. The waste material and AMD released from the spoil heaps were strongly acidic (рН<4.5) due to continuous oxidation of sulfides and subsequent releasing of toxic sulfuric acid, as well as the formation of ferric and ferrous iron sulfates [6]. Oxidation of aluminosilicates in clay minerals (predominantly kaolinite and illite) by acidic waters resulted in the formation of toxic aluminum sulfate in soils [2,7]. Leaching of the gangue also led to migration of the potentially hazardous elements to the soil. Besides, the profile of the overlapped soils had specific morphological properties: carbonaceous-humus films on faces of structural units and secondary gypsum neoformations along with admixture of pyritized fragments and carbonaceous particles in soil pore space.

Because of dewatering of abandoned coal mines, in coal mining areas dips and subsidence up to 6 m deep were formed [9]. Changes in moisture conditions led to the development of semi-hydromorphic soils with different grades of gleying. In the soil profiles of mine subsidence areas, muck accumulation and formation of the organogenic horizon were observed.

The key geochemical processes in Greyzemic Phaeozems at mine sites were as follows: (1) acidification and Fe-Al-SO_4_ salinization of soil profile along with the increasing of H^+^ and Al^3+^ ions content; (2) cation exchange, leading to the displacement of Cа^2+^ and Mg^2+^ by Al^3+^, H^+^ and by Fe^2+^ cations in soil ion-exchange complex; (3) accumulation of potentially hazardous elements. That resulted in the formation of technogenic variations of Greyzemic Phaeozems ‒ acidified, compacted, carbonized, base-unsaturated, and salinized soils.

### 2.2 Sampling procedure

Sampling of soils, waste dump material, natural superficial waters, and AMD was performed at twо abandoned spoil heaps of Moscow brown coal basin: Smirnovskaya-6 (the key site «Кireevsk) and Skuratovskaya-6 (the key site «Tula») and adjacent territories.

Soil samples were taken from the central part of each genetic horizon and in 8 soil pits (4 for each key site) down to 110–150 cm. For each horizon, three subsamples were collected from different walls of the pit to form a composite sample. Reference soils were sampled within 500 m from the spoil heaps. Soils with technogenic transformations were sampled in areas of the deluvial-proluvial deposits and the release of AMD. Two soil pits at the key site «Tula» were located in the mine subsidence area.

Waste material was sampled by drilling down to a depth of 100 cm at the foothill of the Smirnovskaya-6 waste dump and the slope of the Skuratovskaya-6 waste dump.

Natural surface waters samples were taken at 4 locations: from the ponds in karst holes (2 samples) and rivers (2 samples) at a distance of about 500 m from the spoil heaps. The sampling of AMD was performed at 3 points of its release (from waterlogged reservoirs), at the foothills of the waste dumps.

### 2.3 Laboratory methods

AMD and superficial waters were sampled into 500 mL chemically inert plastic containers. The containers were filled up to the lid to avoid the degassing of the water and were placed into a portable refrigerator at 4 °C. The samples of water and AMD were filtered through 0.45-μm PVDF filters (MillesHV, Millipore) and analyzed in the laboratory using high-performance liquid chromatography (HPLC) within 24 h after sampling.

The collected samples (*n* = 100) of waste dumps, technogenic deposits, and soils, were air-dried at temperature <40 °C and were crushed to a particle size of 1 μm for physico-chemical analyses. Prepared bulk samples were stored in special sealed plastic containers. Fresh soil and technogenic deposits samples were used for soil solutions displacement. Soil solutions were displaced by ethanol (Ischtscherikow-Komarova method) [4].

The samples of soil and technogenic deposits at natural moisture were sieved through a 3 mm mesh sieve, then placed in plastic tubes with an inside diameter of 4 cm and 100 cm in height to a bulk density of 1–1.2 g cm^−3^ for soil solution extraction by displacement with ethanol according to Ischtscherikow-Komarova method [4]. Soil solutions (20–40 mL) were collected in chemically inert plastic containers. The testing of the solution for alcohol was made organoleptically. The measurements of each sample were performed in one replicate.

Electrical conductivity (EC) of soil solutions was measured using the conductometer SevenEasy S30 (Mettler Toledo, Switzerland). Anions (Cl^−^, SO_4_^2−^) and cations (Ca^2+^, Mg^2+^, K^+^, Na^+^) in soil solutions were measured by HPLC using a Styer chromatograph (Aquilon, Russia). The results of measurements of soil solutions are given in mmol_c_ dm^−3^. The calculation of the ionic ratios, as well as their proportion in the sum of anions and cations, was performed for ion concentrations, expressed in mmol_c_ dm^−3^. The content of H^+^ and Al^3+^ ions in soil solutions (the sum of H^+^ and Al^3+^ is equal to titratable acidity) was determined by titration to pH 8.2 using a 0.01 М NaOH solution. The total alkalinity of soil solutions was determined by acid-base titration using a 0.01М H_2_SO_4_ solution to pH 4.4. [5]. Total mineralization (in mg L^−1^) was evaluated by summarizing concentrations of all determined elements.

After the displacement of the soil solution, soil samples were removed from the columns. These samples were used to determine the exchange cations. Exchangeable cations (Cа^2+^, Mg^2+^, H^+^, Al^3+^) were determined in bulk soil samples by extraction with 1 M KCl solution at a ratio soil:solution as 1:2.5. The content of exchangeable Ca^2+^ and Mg^2+^ in KCl-extracts was determined by titration using a 0.05М EDTA solution [5]. Exchangeable acidity (the sum of H^+^ and Al^3+^) was released upon exchange by a buffered 1 M KCl solution at the soil to solution ratio of 1:2.5.

The suspension was shaken and filtered. The content of acidic components was determined in the filtrate by titration to pH 8.2 using a 0.01 М NaOH solution [5]. Hydrolytic acidity was measured upon exchange with 1М CH_3_COONa solution at the soil to solution ratio of 1:2.5.

The content of water-soluble and exchangeable (KCl-extracts) Fe^2+^ and Fe^3+^ in soil and soil solutions was measured by UV/Vis spectrophotometry with α-α-dipyridile using Odyssey DR 2000 spectrophotometer (Hach, USA). The pH-values of soil solutions and KCl-extracts were measured by the potentiometric method using Expert 001 ionometer (Econics Expert, Russia).

The total content of C_org_ in soil was determined by K_2_Cr_2_O_7_ oxidation method.

The grain size distribution in soil and technogenic deposit samples was quantified using Analysette 22 MicroTec plus (Fritsch, Germany) laser particle sizer. Samples were pre-treated for analysis by dispersing with 4% Na_4_P_2_O_7_ without H_2_O_2_ oxidation of organic matter. The particle-size classes were defined in accordance with the Russian conventional fraction groups [1].

The total content of macroelements (Fe, Si, Al, Ca, Mg, Ti, P, K) and microelements (Mn, V, Cr, Ni, Zn, Pb, Sr) in technogenic deposits and soils was measured using WDXRF SPECTROSCAN MAKS-GV spectrometer (Spektron, Russia) operating on the proprietary QAV software.

The total content of sulfur in technogenic deposits and soils was measured using portative analyzer WDXRF «Olympus Innov-X Delta» (Delta-X, USA). For XRF analysis, air-dried soil samples were crushed manually in an agate mortar to a particle size of ≤71 μm. The samples were pressed into cups 3 mm deep and specially made from boric acid.

Calculation of the results of soil, technogenic deposits and waste material analyses was done on the basis of oven-dried (at 105 °C) soil mass.

## CRediT Author Statement

**Alexander S. Kostin:** Conceptualization, Investigation, Writing - Original draft preparation, Writing - Reviewing and Editing; **Pavel P. Krechetov:** Methodology, Data Curation, Supervision; **Olga V. Chernitsova:** Software, Visualization; **Elena V. Terskaya:** Validation, Provision of resources.

## Declaration of Competing Interest

The authors declare that they have no known competing financial interests or personal relationships which have, or could be perceived to have, influenced the work reported in this article.
